# Effects of Azithromycin on the Functioning of the Food Web in Freshwater Plankton

**DOI:** 10.3390/jox15050145

**Published:** 2025-09-10

**Authors:** Anita Galir, Dubravka Špoljarić Maronić, Filip Stević, Tanja Žuna Pfeiffer, Fran Prašnikar, Nikolina Bek, Eva Penava, Petra Križevac

**Affiliations:** Department of Biology, Josip Juraj Strossmayer University of Osijek, Ulica Cara Hadrijana 8/A, HR-31000 Osijek, Croatia; agalir@biologija.unios.hr (A.G.); fstevic@biologija.unios.hr (F.S.); tzuna@biologija.unios.hr (T.Ž.P.); fran.prasnikar@biologija.unios.hr (F.P.); nbek@biologija.unios.hr (N.B.); eva.penava@biologija.unios.hr (E.P.); petra.krizevac@biologija.unios.hr (P.K.)

**Keywords:** phytoplankton, zooplankton, rotifers, antibiotic, freshwater, Danube

## Abstract

High doses of the antibiotic azithromycin in freshwater environments can impact planktonic organisms at both the individual and community levels, influencing interactions at the base of the food web. This study investigated the effects of azithromycin on the natural rotifer community feeding on phytoplankton from a eutrophic water body and its potential impacts on rotifer fitness (impaired mastax movement: slow, irregular or reduced frequency), grazing and mortality following acute exposure. The natural plankton community was exposed to three azithromycin concentrations based on the EC_50_ value (EC_50_, 1/2 EC_50_ and 1/3 EC_50_) and assessed at different exposure times (24, 48 and 72 h) in the microcosm experiments. The results showed that all azithromycin concentrations reduced the fitness of the rotifers, as indicated by impaired mastax movement and/or slow, irregular or reduced movement frequency. Impairment of mastax movement altered rotifer grazing and the abundance of phytoplankton. The rotifers in the control group suppressed abundant phytoplankton growth, suggesting that azithromycin impairs interspecific interactions between plankton species. Rotifer mortality occurred at 48 h after azithromycin exposure in all treated samples. These findings show that the effects of azithromycin can be observed at different trophic levels, affecting both phytoplankton and zooplankton through altered biotic interactions and suppressed grazing.

## 1. Introduction

Antibiotic residues have been found in doses ranging from nanograms to micrograms per litre in freshwater bodies around the world [1]. This raises concerns about the impacts of antibiotics in the environment, particularly on non-target organisms [2,3]. The main sources of antibiotic pollution are wastewater treatment plants, livestock farms and hospitals [4], which do not adequately purify their wastewater from pharmaceuticals [5,6]. Although some antibiotics can be efficiently removed in wastewater treatment plants, others remain in wastewater and may pose a high environmental risk. One of the most commonly used antibiotics in veterinary and human medicine at present is azithromycin, a semi-synthetic macrolide antibiotic used to treat infectious diseases of the soft tissues and respiratory tract [7,8]. Since neither animals nor humans fully metabolise antibiotics, 30–90% of the antibiotics administered can be excreted in urine and faeces and enter the environment via the sewage system [5]. The rapidly growing food industry, specifically aquaculture, also utilises large amounts of antibiotics for purposes such as regulating crop health and increasing productivity [9]. Due to the incomplete metabolism and degradation of antibiotics, as well as their insufficient removal through wastewater treatment plants, antibiotics accumulate in the water column and sediment [10]. Despite their widespread use, the fate of antibiotics in the environment is still poorly understood [7,8].

How long antibiotics remain in the environment depends on the environmental conditions; for example, the half-life of azithromycin under aerobic conditions is 82 days, while no degradation was observed under anaerobic conditions [7]. The ecotoxicological effects of antibiotics depend on their concentration and seasonal use, associated with their production, consumption and excretion [11]. Polianciuc et al. [12] found that antibiotic concentrations are higher in the warmer seasons, while Mackuľak et al. [13] detected increased antibiotic concentrations during the winter months—when there is often an increase in inflammatory diseases. Although both studies were conducted in wastewater treatment plants, which discharge their effluents into aquatic systems, the discrepancy in their results might be due to differences in the pharmaceuticals analysed.

In freshwater environments, the food web is formed by planktonic organisms as primary producers (phytoplankton) and primary consumers (zooplankton). Their population dynamics and relationship to each other determine the productivity at higher trophic levels. In addition, different species of both groups contribute uniquely to the carbon, nitrogen and phosphorus cycles in freshwater. In a phytoplankton community, desirable species (e.g., green algae, diatoms) generally support healthy ecosystems, while the development of harmful species—especially cyanobacteria—can produce toxins and cause harmful algal blooms leading to oxygen depletion and fish mortality. Various studies have been conducted to investigate the effects of antibiotics on phytoplankton, but there is still a lack of knowledge about the toxicological effects of antibiotics, including how they vary in nature [14,15]. Azithromycin can have a dual effect on microalgae: at low concentrations (<1 μg/L), it can induce photosynthetic and antioxidant activities while, at high concentrations (5–100 μg/L), it inhibits algal growth [15]. Azithromycin has been shown to promote oxidative damage to membranes and DNA in phytoplankton by impairing energy dissipation and other defence mechanisms against photodamage and reactive oxygen species (ROS) [14]. The effects of macrolide antibiotics are also species-dependent. While the use of azithromycin in the green algae *Raphidocelis subcapitata* leads to a negative influence on the regulation of energy dissipation in the PSII centres and, thus, to photodamage, the effect of erythromycin on the cyanobacteria *Microcystis flos-aquae* causes significant increase in superoxide dismutase and catalase activities as well as malondialdehyde concentrations, indicating severe oxidative stress [16,17]. In another study, the filamentous cyanobacterium *Cylindrospermopsis* sp. showed a strong negative response, while the abundance of *Microcystis* sp. increased [18]. Most of these studies agree and suggest that azithromycin—which is frequently detected in freshwater environments—could pose a significant ecological risk in real-world environments. It is also possible that pharmaceuticals present in water can induce the release of biogenic amines by stressed zooplankton, which subsequently stimulate algal growth [19].

Not all phytoplankton species are equal in terms of nutritional value: some species are more easily consumed by zooplankton and support healthy food webs, while others are inedible or even toxic, disrupting energy flows and impacting fish populations. As all parts of the food web are intertwined, zooplankton species are considered important mediators in the food web. Antibiotics have been shown to accumulate to significant levels in zooplankton tissues with constant exposure, which are then transferred to higher trophic levels such as larger invertebrates, fish larvae and adult fish [20]. It has been shown that the addition of probiotics to the diet of rotifers increase their growth rate and average swimming speed [21], which is important not only for food intake but also for escaping from predators. Furthermore, the rotifer microbiome is more strongly influenced by a combination of host ecology and habitat influence than by phylogenetic host distances [22]. Thus, we can assume that the effects of antibiotics—when present in the environment—occur at the community level and not just at the individual level. The imbalance in the oxidative stress–antioxidant system induced by antibiotics can cause ecological cascades [23,24,25], leading to the dominant growth of more tolerant species. This could affect the trade-off in rotifers, as their reproductive rate and average lifespan change when they are fed with different diets, even under sufficient feeding conditions [26]. Apart from some zooplankton species commonly used in ecotoxicological studies, there are few studies on other zooplankton species, especially rotifers [27]. The use of antibiotics in rotifers leads to impaired neurotransmission, inhibition of digestive enzymes and, ultimately, a reduction in body size [28]. The effects of antibiotics are also related to the trophic status of water bodies, as the antibiotic concentration in zooplankton is positively related to the total nitrogen (TN) and total phosphorus (TP) concentrations in the water column. In eutrophic freshwater systems, total phosphorus and total nitrogen concentrations may increase the risk of bioaccumulation of various antibiotics in zooplankton feeding on phytoplankton, as they are able to accumulate antibiotics and form large populations under elevated nutrient concentrations [29,30,31]. As with phytoplankton, there are two conclusions regarding the effects on zooplankton, which are related to the type of antibiotic. Even within the group of macrolide antibiotics, rotifers have been reported to be particularly affected by erythromycin, whereas the effects of clarithromycin were less pronounced in chronic toxicity tests [32].

Considering that the Danube catchment area covers more than 800,000 km^2^ (or 10% of the European continent) and is home to more than 80 million people whose activities inevitably impact the main river and its tributaries [33], it can be assumed that the footprint of antibiotic use on its freshwater environment is significant. The aim of our study was to test the impact of azithromycin on the natural rotifer community feeding on the natural phytoplankton community of the Danube floodplain lake, and to investigate its possible effects on the fitness and mortality of the organisms after acute exposure. To test these changes, we conducted a microcosm experiment in which the natural plankton community was exposed to three different azithromycin concentrations. We hypothesised that (i) all azithromycin concentrations will affect rotifer fitness (disturbed mastax movement: slow, irregular or reduced frequency); (ii) altered rotifer fitness will lead to reduced grazing and increased phytoplankton abundance, especially at higher azithromycin doses; (iii) mortality in rotifers will increase earlier at high azithromycin concentrations; and (iv) all azithromycin concentrations will alter natural food web dynamics.

## 2. Materials and Methods

### 2.1. Sampling and Analysis of Limnological Parameters

**Figure 1:**
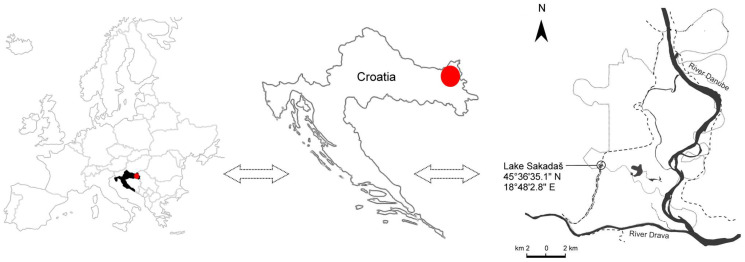
Map of the study area. Location of the Kopački Rit Nature Park (red dot) in Croatia and detailed map of the floodplain.

The sampling was carried out in May 2025 in the floodplain of the Kopački rit Nature Park in Croatia (Figure 1), in the south-eastern part of Europe. The water and plankton samples were taken from Lake Sakadaš, which is the deepest water body (average depth 7 m; area 0.12 km^2^) [34]. The main water supply of Kopački rit comes from the Danube and, to a lesser extent, from the Drava. The area is a Natura 2000 site and is part of the UNESCO Biosphere Reserve Mura-Drava-Danube.

For the experimental setup, 180 L of surface water was filtered through a 10 μm plankton net for phytoplankton analysis, 120 L for zooplankton through a 25 μm plankton net, and 1.5 L of lake water was taken and later used as a medium for the plankton exposure. For the qualitative assessment of phytoplankton and zooplankton, 10 L of lake water was filtered through corresponding plankton nets and fixed in formaldehyde (4%). For quantitative analysis of phytoplankton, unfiltered samples were taken and fixed in acidified Lugol’s solution.

Environmental parameters, including water temperature (WT, °C), dissolved oxygen (OC, mg/L), oxygen saturation (OS,%), pH and conductivity (Cond, μS/cm), were measured at the site using a portable three-channel multimeter (HQ4300, Hach Company, Loveland, CO, USA). An echo sounder was used to measure the water depth (WD, m), while transparency (SD, m) was measured using the Secchi plate. One surface water sample (1 L) was collected for chlorophyll-a analysis (Chl-a) and another (1 L) for chemical analysis of the water.

The water for Chl-a analysis was filtered under vacuum through an MN GF-3 filter paper (Macherey-Nagel, Düren, Germany), pigments were extracted in acetone and the absorbance was measured after 24 h using a Boeco spectrophotometer S-200 (Boeco, Hamburg, Germany). The Chl-a concentration was determined according to the methods reported in [35,36]. The chemical analysis of the water included measurement of the concentrations of nitrite (NO_2_-N, mg/L) [37], nitrate (NO_3_-N, mg/L) [38], ammonia (NH_4_^+^, mg/L) [39], total phosphorus (TP, P mg/L) [40] and total nitrogen (TN, N mg/L) [41], which were carried out in an accredited laboratory of the Institute of Public Health.

### 2.2. Experimental Design

In the laboratory, the lake water was visually inspected to remove debris. The sample was homogenised using a hand stirrer and examined under an Olympus BX51 (Tokyo, Japan) microscope to calculate the total number of zooplankton individuals per ml. It was calculated that 5 mL of the homogenised sample contained 500 rotifer individuals. Of this homogenised sample, 60 mL was separated from the rest of the lake water sample. The water sample (1.5 L) was filtered under vacuum through an MN GF-3 filter (Macherey-Nagel, Düren, Germany), and the filtrate obtained was used as the medium for the experiment in both the test and control groups. In particular, 45 mL of medium was added to each Petri dish together with 5 mL of zooplankton sample (Table 1). To condition the community, the samples were placed for 24 h at a constant temperature (18–20 °C) with a natural light source.

To prepare a starting solution with a concentration of 5 mg/mL, 500 mg azithromycin (Makromicin, JGL d.d., Rijeka, Croatia) was dissolved in 100 mL distilled water. The azithromycin test solutions were prepared by adding different volumes of the starting solution to a Petri dish and diluting to 50 mL with distilled water. Concentration C corresponds to the EC_50_ value [27,42], while concentration B corresponds to 1/2 EC_50_ and concentration A corresponds to 1/3 EC_50_.

Phytoplankton, which is a food source for zooplankton in the natural environment, was also added to the filtrate. Phytoplankton samples were centrifuged (3000 rpm; 15 min) and resuspended in filtered lake water to ensure that as little of the substance as possible was included in the feeding experiment. The prepared phytoplankton suspension was further diluted with filtered lake water to achieve the desired cell concentration, which was determined by counting the cells pre-treated with 4% formaldehyde in a Bürker-Türk chamber under an inverted microscope (Zeiss Axiovert 5, Carl Zeiss, Jena, Germany).

After conditioning the zooplankton for 24 h, antibiotic suspensions and phytoplankton (250,000 cells/mL) were added to each Petri dish (Table 1) to obtain a total volume of 50 mL. All exposure categories and a control group were performed in triplicate.

### 2.3. Plankton Community Analyses

For the qualitative analysis of rotifers, a uniform sample was used in which at least 300 rotifers were counted using an Olympus BX51 microscope (Tokyo, Japan). Prior to microscopy, the sample was centrifuged at 2500 rpm and the species were identified using specialised keys. Data are expressed as ind/L.

To analyse the phytoplankton size classes as a food source, the individuals were identified to species level using standard identification keys. Quantitative assessment of the phytoplankton was based on [43], and cell measurements were performed with an inverted microscope (Zeiss Axiovert 5, Carl Zeiss, Jena, Germany), Zeiss Axiocam 208 and the ZEN 3.11 blue edition software (Carl Zeiss, Jena, Germany). The abundance of phytoplankton was estimated according to the methods described in the national methodology and are expressed in cells/L [44].

The experiment was conducted for 72 h. The abundance of phytoplankton was monitored every 24 h together with the fitness and mortality of rotifers. Rotifers were examined under a microscope (×100 magnification), observing mastax movement (normal: rhythmic, active grounding motion; disturbed: slow, irregular or reduced frequency; absent: no visible mastax activity) to detect any toxicity affecting rotifer fitness. At least 10 individuals from each sample were observed and the frequency of mastax movement of each individual was monitored for 1 min. Mortality was also visually analysed and recorded, with a maximum mortality of 10% in the controls for the test to be considered valid [45].

### 2.4. Statistical Analyses

An independent t-test was performed to test the difference in phytoplankton abundance between the groups treated with different concentrations of azithromycin. Significant differences were considered at *p* < 0.05. The same procedure was used to test the difference in fitness and mortality of the zooplankton groups. Analyses were performed in STATISTICA v. 14.0.1.25 (TIBCO Software Inc., Palo Alto, CA, USA). A web-based Sankey diagram generation programme, SankeyArt version 1.4 was used to create a Sankey diagram to visualise the observed fitness and mortality data of the rotifer community after exposure to different azithromycin concentrations.

## 3. Results

### 3.1. Limnological Parameters

The measured environmental parameters corresponded to the long-term data of typical values measured in this area (Table 2). The water temperature corresponded to the sampling performed in late spring, and high oxygen levels were recorded. In addition, the TN and TP values indicate a water body with an elevated trophic state, which is characteristic of the Danube floodplain waters.

### 3.2. Plankton Community

A total of 11 rotifer species were found in the zooplankton at the time of sampling (Table 3). Some of these species feed on small prey such as bacteria and minute algae (*Keratella tecta*, *Pompholyx sulcata*), but rotifers that feed on larger organisms (*Polyarthra vulgaris*, *Synchaeta* sp.) were also present in the community. This indicates a diverse food web as different types and sizes of prey were present in the environment, including species that also feed on animal food such as *Trichocerca* sp. (Table 3).

The phytoplankton community (55 taxa in total) comprised cyanobacteria, cryptophytes, euglenophytes, chrysophytes, diatoms and chlorophytes of various size classes (Table 3). Algae with a size of 5–10 µm were the most abundant (39% of the total phytoplankton abundance) and diversified (22 taxa), especially the cryptophycean flagellate *Plagioselmis nannoplanctica* (27%) and, to a lesser extent, the chlorophyte *Raphidocelis danubiana* and the euglenophyte *Trachelomonas volvocina*. Representatives of the size fraction > 50 µm were almost as common, with only 9 taxa accounting for 35% of the total phytoplankton abundance, particularly the filamentous cyanobacteria *Planktolyngbya limnetica* and *Pseudanabaena limnetica*.

**Figure 2:**
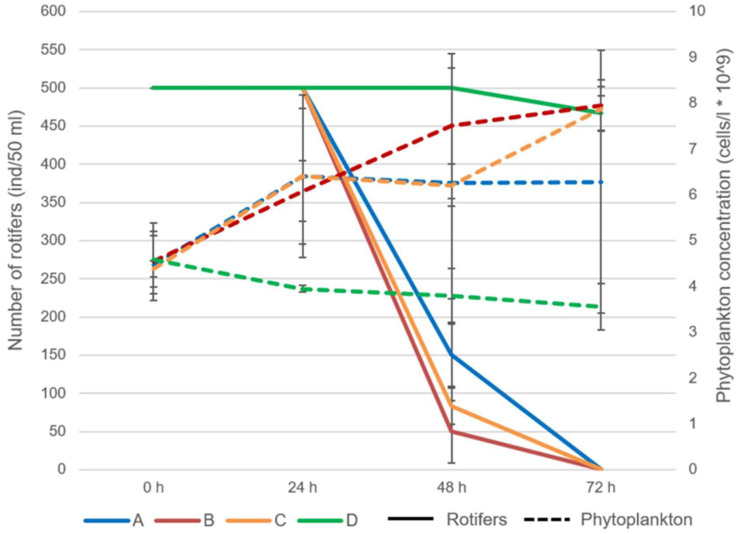
The abundance of rotifers and phytoplankton during the experiment with acute exposure to azithromycin (Abbreviations: A: concentration 1; B: concentration 2; C: concentration 3; D: control).

**Figure 3:**
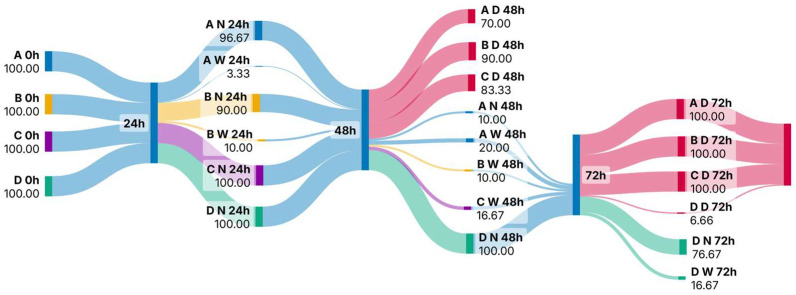
Sankey diagram showing the fitness and mortality rates of rotifers during acute exposure to azithromycin (Abbreviations: A: concentration 1; B: concentration 2; C: concentration 3; D: control; N: normal mastax activity; W: weakened mastax activity; D: dead).

Throughout the experiment, zooplankton mortality increased in the azithromycin-treated samples (Figure 2). At the beginning of the experiment and within 24 h of exposure, all individuals examined were alive (Figure 3). At 48 h after exposure, the mortality of rotifers increased from 70% at concentration 1 to 90% at concentration 2. At 72 h after exposure, all examined individuals were dead (Figure 2).

The phytoplankton abundance increased significantly in the treated samples B (t-value: 10.8; *p* < 0.001) and C (10.79; *p* < 0.001) when compared to the control group at 72 h after exposure, at which point the mortality of rotifers reached 100%.

The fitness of the individuals also decreased over time (Figure 3). Within 24 h of exposure, the fitness of individuals began to change and the percentage of individuals with altered mastax activity ranged from 3.3 to 10%. Significant differences between the different concentrations were observed in terms of both the fitness and mortality of rotifers. At 24 h and 48 h after exposure, the fitness of rotifers changed between samples A and B with t-value: −3.28, *p* < 0.03 and t-value: 5.51, *p* < 0.005, respectively. From then on, the fitness of the individuals decreased until mortality at the end of the experiment (Figure 3). A significant difference in rotifer mortality was observed at 48 h after exposure between samples A and B (t-value: 13.59; *p* < 0.001). At the same time point, the mortality in all samples treated with azithromycin differed significantly from the control group (A—t-value: 60, *p* < 0.001; B—t-value: 15.42, *p* < 0.001; C—t-value: 24.7, *p* < 0.001, respectively). Mortality in the control group was less than 8% throughout the experiment (Figure 2).

## 4. Discussion

The waters of the Danube are eutrophic along their entire course [49]. The long-term monitoring of the waters of the Kopački Rit Natural Park shows no exception, and the measured concentrations of TP and TN correspond to this trophic state. At the time of sampling, the environmental parameters were typical for late spring in this area. The WT was typical for the spring season, supporting the optimal growth of phytoplankton and lying in a range in which many species grow [50]. The oxygen concentrations were correspondingly high. The high Cond values measured are common in this area, due to the sediment load from the main river and resuspension during hydrological changes [49].

In addition to the well-developed phytoplankton community, zooplankton—including rotifers—are also more well developed in eutrophic freshwater ecosystems when compared to those with lower trophic levels [51,52]. Rotifers are sensitive to environmental parameters, and their species diversity and abundance depend on the habitat type and the trophic state of the ecosystem [52]. In eutrophic waters, the great diversity of algae can provide diverse food sources that allow for the development of different niches, as was the case in our community. In such situations, specialised species that can feed selectively and avoid predators evolve best [53]. This interaction between phytoplankton and zooplankton has been found to be largely influenced by the hydrology of the area where the algivorous rotifers, bacterivores and predators meet [54]. Previous studies have shown that high doses of antibiotics can affect plankton at both individual and community levels, although these concentrations rarely occur in natural ecosystems [55]. We also did not investigate environmentally relevant concentrations of azithromycin, but the potential impacts of its introduction on non-target organisms and the freshwater food web have been recorded. This is particularly important in the context of climate change, as the average annual temperature has been shown to have a detectable effect on antibiotic concentrations [56].

One of the parameters measured in our experiment was the function of the mastax (a modified pharynx), which is used for food processing in rotifers. Depending on the type of food favoured, the mastax processes food by grasping, piercing, crushing or scraping [57]. The feeding behaviour of rotifers is frequently used in ecotoxicology as it is a quick and sensitive endpoint for observing the impact of a pollutant, as the energy distribution in rotifers depends mainly on feeding [58]. In studies on various anthropogenic pollutants, rotifers are often used as they are considered more sensitive to anthropogenic substances than other zooplankton groups (e.g., Cladocera) [59]; however, most studies have utilized farmed monocultures and only a few have used natural rotifer communities with different species. Our results show that the fitness of individual rotifers within the community started to change as early as 24 h after exposure with an altered mastax activity of up to 10%, which continued to increase at 48 h after exposure. Similar studies have revealed several key aspects that can be associated with reduced mastax activity, such as increased ROS levels that inhibit rotifer feeding [23,28], impair neurotransmission and inhibit digestive enzyme activity [28], even at the lowest toxin concentrations [58].

Reduced mastax activity is related to the movement of the corona, as these organs are interconnected and the corona facilitates both locomotion and food intake [46,48]. In our study, all individuals that showed reduced mastax movement also showed reduced body movement, as has been observed elsewhere with reduced hopping frequency of zooplankton species under the influence of antibiotics and herbicides [60]. Similar results have also been observed with other anthropogenic agents, where control individuals swam in a highly sinuous manner compared to treated rotifers [21], and reduced ciliary activity was observed in treated individuals [61]. This could be due to nutritional stress as well as energy balance, as rotifers have the physiological ability to regulate their resource consumption and suppress their activity when conditions change [62]. All rotifers tested died within 72 h of exposure to azithromycin, which has also been observed elsewhere following antibiotic treatment [42].

The uptake and filtration of antibiotics in the aquatic environment through the plankton food web is a complex component of the antibiotic cycle, and antibiotic bioaccumulation is strongly correlated with the biomass of phytoplankton and zooplankton [20]. For phytoplankton, the opposite results were observed regarding the effects of antibiotics. Azithromycin can stimulate algal growth at low concentrations while, at higher doses it can inhibit algal growth, which increases with exposure time and azithromycin concentration [15]. At low antibiotic doses, the abundance and biomass of phytoplankton as well as the contents of pigments, lipids, proteins and carbohydrates increase [63], which is closely related to the availability of nutrients in the environment [64]. However, the response of phytoplankton to the same dose has been shown to be species-specific and species can behave differently, suggesting that the antibiotic treatment also affects plankton at the community level [18]. In our study, the abundance of rotifers decreased and the phytoplankton concentration increased with exposure to azithromycin in all treatments. However, our treatments were in the range of higher antibiotic concentrations reported in the literature and we expected to inhibit algal growth. Although the mechanisms for increasing phytoplankton populations through the addition of antibiotics are known, the species-specific biotic interactions associated with the plankton food web also need to be considered. The inhibition of feeding activities and reduced grazing by rotifers may have further facilitated the increase in phytoplankton, as rotifers can effectively suppress phytoplankton growth [65].

In a study on the effects of antibiotics on organisms in the lower food web, shifts in the composition of microbes and phytoplankton significantly influenced changes in zooplankton [18]. In the community we studied, the recorded phytoplankton species served as high-quality food for rotifers. The abundant development of phytoplankton in the size range of 5–10 μm—especially cryptophycean flagellates—supports the development of rotifers, as they are small and thus fit within the ingestion range of rotifers [48]; have high nutritional quality as they are rich in polyunsaturated fatty acids, amino acids and sterols [66]; have a soft cell wall and, thus, are easily digestible compared to diatoms [67]; and have high growth rates that ensure a continuous food supply in natural systems [68]. Filamentous cyanobacteria, which belong to the larger size category (>50 μm), were also present in large numbers in our investigated phytoplankton community, but are inedible for zooplankton. While *Syncaheta* species can ingest cyanobacteria, this refers only to unicellular or small cyanobacteria [69], and even these show high growth and reproduction rates when fed with cryptophytes [70]. Moreover, the presence of *Lecane* rotifers feeding on cyanobacterial exopolysaccharide mucilage increases the dispersion of cyanobacterial trichomes, thereby increasing the availability of filaments for other grazers [71]; this can also be assumed for mucilaginous colonial cyanobacteria with small cell size in our experiment. Due to the high-quality food sources supporting the abundant rotifer community in natural ecosystems, the change in rotifer fitness cannot be attributed to low food supply or experimental error. The addition of azithromycin at all concentrations tested interfered with regular biotic interactions and reduced rotifer feeding, which remained effective in the control groups throughout the experiment. The effects of azithromycin were thus evident at different trophic levels, affecting both phytoplankton and zooplankton through altered biotic interactions and suppressed grazing.

## 5. Conclusions

Acute exposure of the natural plankton community of a eutrophic water body to azithromycin led to diverse effects. The fitness of rotifers began to decline at 24 h after exposure in all treated samples at different concentrations, as indicated by impaired mastax movement and/or slow, irregular or reduced movement frequency. Impaired mastax movement affected rotifer grazing and, at the same time, phytoplankton began to proliferate. It was expected that mortality of individuals would occur earlier at higher azithromycin doses, but mortality occurred at 48 h after azithromycin exposure in all treated samples. While all concentrations altered rotifer fitness and phytoplankton growth, rotifers in the control group suppressed abundant phytoplankton growth, showing that azithromycin affects interspecific interactions between plankton species. Although we used azithromycin concentrations that are rarely observed in nature, this study emphasises the potential of azithromycin to affect non-target organisms and highlights the potential hazards if azithromycin is not properly removed from wastewater.

## Figures and Tables

**Table 1:** Experimental setup and the volumes added such that the final volume of each sample was 50 mL (Abbreviations: A: concentration 1; B: concentration 2; C: concentration 3; D: control; the numbers next to the letters (A, B, C and D) show the number of replicates).

Antibiotic Concentration (mg/mL)		Added Volume (mL)				Fitness/Mortality		
		Filtrate	Zooplankton	Phytoplankton	Antibiotic			
1.	0.047	43.6	5	0.93	0.47	A1	A2	A3
2.	0.07	43.4	5	0.93	0.7	B1	B2	B3
3.	0.14	42.7	5	0.93	1.4	C1	C2	C3
Control		45	5	0.93	-	D1	D2	D3

**Table 2:** Limnological parameters measured during sampling in the Kopački rit Nature Park in 2025. Abbreviations: SD—transparency; WT—water temperature; OC—dissolved oxygen concentration; OS—oxygen saturation; Cond—electrical conductivity; NO_2_-N—nitrite; NO_3_-N—nitrate; NH_4_^+^—ammonia; TP—total phosphorus; TN—total nitrogen; Chl-a—chlorophyll-a.

Parameter	Value
SD (m)	0.89
WT (°C)	18.6
OC (mg/L)	8.67
OS (%)	93.7
pH	7.8
Cond (μS/cm)	816
NO_2_-N (mg/L)	0.002
NO_3_-N (mg/L)	0.058
NH_4_^+^ (mg/L)	0.022
TP (mg/L)	0.089
TN (mg/L)	0.706
Chl-a (μg/L)	37.8

**Table 3:** Natural rotifer community used in the experiment, assigned based on the available phytoplankton food size categories [46,47,48].

Recorded Rotifer	Preferred Rotifer Prey, Size and Type	Recorded Phytoplankton as a Probable Food Source
Species		
*Keratella tecta, Keratella cochlearis*, *Lecane lunaris*	Bacteria-detritus suspension; not larger than several µm	<2 μm; *Aphanocapsa delicatissima* (average colony size 11.29 μm), *Merismopedia tenuissima* (average colony size 18.43 μm)
*Epiphanes* sp., *Lepadella patella*, *Pompholyx sulcata*, *Ptygura* sp.	Bacteria-detritus suspension and minute algae typical of eutrophic environments	5–10 μm; *Aulacoseira pusilla*, *Chrysococcus rufescens, Coelastrum microporum*, *Cryptomonas ovata*, *Cyclotella* sp., *Desmodesmus abundans*, *Kephyrion rubri-claustri*, *Lagerheimia genevensis*, *L. wratislawiensis*, *Lemmermannia tetrapedia*, *Mallomonas* sp., *Monoraphidium minutum*, *Oocystis lacustris*, *Phacotus lenticularis*, *Plagioselmis nannoplanctica*, *Pseudodidymocystis inconspicua*, *P*. *planctonica*, *Raphidocelis danubiana*, *Stephanodiscus* sp., *Tetradesmus dimorphus*, *T. lagerheimii*, *Tetraëdron minimum*, *Tetrastrum glabrum*, *T. staurogeniiforme*, *Trachelomonas volvocina*
*Keratella quadrata*	Phytoplankton below 20 μm; sometimes bacteria and detritus	10–20 μm; *Actinastrum hantzschii*, *Ankistrodesmus falcatus*, *Cryptomonas erosa*, *C*. *marssonii*, *M. arcuatum*, *Monoraphidium contortum*, *Plagioselmis lacustris*, *Skeletonema potamos*, *Trachelomonas oblonga*
*Polyarthra vulgaris*, *Trichocerca elongata*	Phytoplankton ranging 20–30 μm	20–30 μm; *Ankyra* sp., *Centritractus belonophorus*, *Fragilaria tenera*, *Gonium pectorale*, *Koliella longiseta*, *Monactinus simplex*, *Mucidosphaerium pulchellum*, *Nitzschia paleacea*, *Pediastrum duplex*, *Schroederia spiralis*, *Nitzschia* sp.
*Synchaeta* sp.	Maximum food particle size 50 μm	30–50 μm; -
Generally inedible for rotifers		>50 μm; *Anabaena* sp., *Aulacoseira granulata*, *Asterionella formosa*, *Dinobryon divergens*, *Nitzschia acicularis*, *Phormidium* sp., *Planktolyngbya limnetica*, *Pseudanabaena limnetica*, *Ulnaria acus*

## Data Availability

The original contributions presented in this study are included in the article. Further inquiries can be directed to the corresponding author(s).
